# An Evaluation of the Safety Effectiveness and Cost of Autonomous Vehicles Based on Multivariable Coupling

**DOI:** 10.3390/s23031321

**Published:** 2023-01-24

**Authors:** Hong Tan, Fuquan Zhao, Wang Zhang, Zongwei Liu

**Affiliations:** 1 State Key Laboratory of Automotive Safety and Energy, Tsinghua University, Beijing 100084, China; 2 Tsinghua Automotive Strategy Research Institute, Tsinghua University, Beijing 100084, China

**Keywords:** autonomous vehicles, V2X, crash avoidance effectiveness, sensors, atomic technology combination, safety function, life-cycle cost sharing

## Abstract

There is a need for in-depth studies of autonomous vehicle safety that evaluate the effectiveness of safety functions and different “atomic” technology combinations for vehicles and roads. In this paper, we provide a crash avoidance effectiveness evaluation model for autonomous vehicles enabled with different sensor combinations based on multiple variables of 14 different “atomic” sensing technologies on the vehicle side and road side, 52 safety functions, and 14 accident types. Meanwhile, a cost-sharing model is developed based on the traveled distance during the life cycle of vehicles and based on the traffic flow over the life cycle of roads to evaluate the unit cost per km of different “atomic” technology combinations. The results clearly show that the cost increases with the addition of “atomic” sensing technologies on the vehicle side, while an increase in crash avoidance effectiveness decreases. It is necessary to switch to V2X and to introduce roadside “atomic” technology combinations to realize better safety effectiveness at a lower cost for vehicles. In addition, a map that covers the safety effectiveness and cost per kilometer of all “atomic” technology combinations is calculated for decision-makers to select combinations under the preconditions of cost and safety.

## 1. Introduction

Software-defined vehicle (SDV) is the general trend of the automotive industry, with the concept of decoupling software and hardware and creating a new research topic of “atomic” technology combination [1]. In the past, the architectures of autonomous vehicles have realized one function with a set of parts and components. The typical feature was that hardware and software were coupled. With such an architecture, automakers purchase software functions interfaced with hardware, the corresponding hardware cannot be changed. Meanwhile, the hardware cannot be reused, and a specific function is not available without a combination of specific hardware and software, and therefore, the cost is relatively higher. Moreover, there is no space or possibility for vehicles to evolve. New functions and services require the addition of corresponding parts and software. Therefore, future architectures of autonomous vehicles require the decoupling of software and hardware. Hardware would be translated into atoms, which would be orchestrated to realize safety functions. This so-called hardware atomization means translating hardware that has been decoupled with software into technological “atoms” to be revoked by various softwares [1,2,3]. Autonomous vehicles with an architecture in which software and hardware are decoupled would have the following advantages [4]: First, original equipment manufacturers (OEMs) could flexibly replace hardware, since software and hardware are decoupled. Second, hardware could be reused. After the standardization and abstraction of hardware, one hardware could realize several functions, thus, reducing the number of hardware units and saving cost. Third, OEMs could add software to add more functions, without the need of replacing or adding hardware, leaving more space and possibilities for vehicles to evolve. The so-called atomization technologies refer to minimal hardware units of autonomous vehicles, such as LiDAR, cameras, the steering system, and the braking system. Such units are combined and orchestrated to realize the functions of autonomous vehicles [1]. Since the safety and cost of autonomous vehicles would vary based on the “atomic” technology combination, there is a need to study the safety and cost of autonomous vehicles brought by the combination of “atomic” technologies.

Vehicle to everything (V2X) has gradually become an industry-wide consensus, especially in China [5,6], and has generated a new research topic of how to distribute capabilities and costs taking into consideration vehicles and roads. From the perspective of technology, autonomous vehicle technologies are complex and there will always be long-tailed problems. However, V2X can improve the intelligent capabilities of vehicles and provide redundancy. From the perspective of cost, a higher level of intelligence would increase the cost of vehicles, making it hard to promote the uptake of autonomous vehicles, which could fall into the “nobility” vehicles. V2X could reduce the cost of vehicles and could turn autonomous vehicles into a reality by migrating relevant hardware such as cameras, MMW radars, and LiDAR to roads to provide intelligent functions for vehicles [7]. Therefore, the cost of such hardware would be shared by thousands of vehicles and the utilization would also increase. Otherwise, the cost would be woven into the total cost of autonomous vehicles. From the perspective of benefits, autonomous vehicles can only improve themselves and generate local benefits, failing to improve traffic flow. However, V2X would make global improvements and would maximize social benefits. The essence of V2X is to distribute “intelligent capabilities and costs” to vehicles and roads, which requires the analysis of the safety and cost under different combinations of “atomic” technologies.

The safety mechanism of autonomous vehicles is to use different safety functions to avoid accidents and to improve the safety of vehicles. There has been a lot of research on the safety effectiveness of the safety functions of autonomous vehicles [8]. Regarding automatic emergency braking (AEB), most studies have only focused on rear-end crashes. Some studies have mainly focused on pedestrian crashes and cyclist crashes. According to the available evidence, the effectiveness of AEB in avoiding target crashes ranges from 18% to 72% [9,10]. For adaptive cruise control (ACC), the effectiveness of ACC in avoiding a rear-end crash and other related crashes ranges from 12% to 16%, which is a relatively low level [11,12]. For lane keeping assist (LKA) and lane departure warning (LDW), lane departure-related crashes could be avoided by using LKA and LDW, including single crashes, front crashes, sideswipe same direction crashes, and sideswipe opposite direction crashes. The effectiveness of LDW is in the range of 10–48% [13]. The effectiveness of LKA is in the range of 20–51% [14]. For blind spot detection (BSD), according to the related references, the target crash scenario that could be avoided by BSD is the lane-change crash and the crash avoidance effectiveness of BSD ranges between 14% and 58% [15,16]. For connected intersection movement assist (IMA), the intersection crashes that could be avoided by IMA, include straight crossing paths at non-signal (SCP), left turn into the path at non-signal (LTIP), right turn into the path at signal (RTIP), running a red light, and running a stop sign. The crash avoidance effectiveness of IMA is in the range of 23–67% [17,18]. For connected left-turn assist (LTA), the left turn across the path crash could be avoided by using LTA technology. The effectiveness of LTA is in the range of 32% to 60% [19,20]. It should be noted that using V2X to realize connected AEB (C-AEB), connected LKA (C-LKA), connected ACC (C-ACC), and connected LDW (C-LDW) would result in better crash avoidance effectiveness [9,21]. In addition to avoiding collisions, mitigating harm of collision is also a research direction. Parseh and Asplund proposed a collision reconfiguration system that could mitigate harm due to collision by changing where vehicles were hit and how the impact force was directed towards the vehicle [22]. Test scenarios for autonomous vehicles have received a lot of attention. A systematic and data mining approach was developed to extract and generate high-risk precrash test scenarios from the data [23]. Speed and space perception also affects the occurrence of road traffic accidents [24]. Yu developed a binary logit regression model to differentiate the hazardous scenarios of autonomous vehicles [25]. The model was developed to quickly generate various test scenarios by adjusting various traffic scenarios and different severities of quantifiable weather and interference parameters [26].

Under the new development trends of V2X and SDV, the safety effectiveness of autonomous vehicles involves numerous “atomic” technology combinations and the coupling of multiple safety functions, therefore, requiring a method to evaluate the safety effectiveness based on the coupling of multiple variables. As compared with previous research, to evaluate the safety effectiveness of autonomous vehicles, studies should go to a deeper level, namely, evaluate the safety effectiveness of “atomic” technologies including vehicle-side cameras, millimeter-wave (MMW) radar, LiDAR, high-precision positioning units, automotive computing units, the brake-by-wire system, the steer-by-wire system, the on-board unit (OBU) for communication, as well as roadside cameras, MMW radars, LiDAR, roadside units (RSUs) for communication, and computing units [1]. Among these “atomic” technologies, “atomic” sensing technologies are the foundation and the core of securing the safety of autonomous vehicles, and are selected as the research object in this paper. The “atomic” technologies in this paper mainly focus on the sensing system, including cameras, MMW radars, as well as vehicle-side and roadside LiDAR. Only by atomizing technologies can they be recombined. The “atomic” technology combinations mean orchestrating them. Safety effectiveness is reflected by the crash avoidance effectiveness of vehicles. Different types of “atomic” technologies in different positions can enable various types of safety functions of autonomous vehicles, which can avoid a certain proportion of target accidents, thus, generating the comprehensive crash avoidance effectiveness of vehicles. In this paper, we evaluate the safety effectiveness and cost of autonomous vehicles from such a perspective.

In this paper, we provide an “atomic” technology safety effectiveness evaluation model with the coupling of multiple variables based on “atomic” technologies, safety functions, target accident types, and crash avoidance effectiveness, and we provide an “atomic” technology cost-sharing model based on the traveled distance during the life cycle of vehicles and based on the traffic flow over the life cycle of roads. We apply the models to mainly answer the following questions in a systematic manner:

(1) How can the crash avoidance effectiveness of different “atomic” sensing technologies be quantified realizing various safety functions?
(2) What is the crash avoidance effectiveness of the “atomic” sensing technology combinations? What would the life-cycle cost be of the combinations?
(3) What is the “atomic” technology combination to meet the safety requirements at the lowest cost? What is the “atomic” technology combination to feature the highest crash avoidance effectiveness with a certain cost.

Some summarized highlights of the results include: (1) Roadside sensors can result in a higher comprehensive traffic crash avoidance effectiveness and a lower cost to use per kilometer than vehicle-side sensors. (2) The cost would increase with the addition of “atomic” sensing technologies on the vehicle side, while the increase in crash avoidance effectiveness would slow down. (3) It is necessary to switch to V2X and introduce roadside “atomic” technology combinations to realize better safety effectiveness at a lower cost for vehicles. (4) From the perspective of crash effectiveness and lift-cycle cost sharing, the “atomic” technology combinations for V2X would be superior to the “atomic” technology combinations only for vehicles.

## 2. Methodology

**Figure 1:**
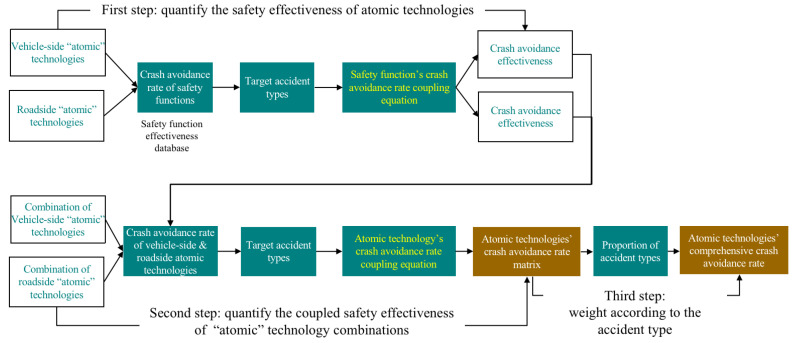
Safety effectiveness multivariable coupling model based on “atomic” technologies, safety functions, accident types, and crash avoidance effectiveness.

To measure the safety effectiveness of autonomous vehicles enabled by the combination of different “atomic” technologies, in this paper, we provide a safety effectiveness evaluation model based on multiple variables of “atomic” technologies, safety functions, accident types, and crash avoidance effectiveness, as shown in Figure 1 and Equations (1)–(5). There are three steps. The first step is to quantify the safety effectiveness and target accident types of each “atomic” technology. It is necessary to identify multiple safety functions that can be realized by the “atomic” technology. Then, we calculate corresponding crash avoidance effectiveness for the target accident types of the “atomic” technology, realizing several safety functions, as shown in Equation (5). The second step is to quantify the target type of accidents and corresponding coupled crash avoidance effectiveness of the “atomic” technology combinations. Each “atomic” technology in the combination has target accident types and corresponding avoidance effectiveness. For the accident type that is listed as the target by multiple atomic technologies, the comprehensive collision avoidance effectiveness can be calculated by using Equation (4). The third step is to weigh the type of accident and the crash avoidance rate of the “atomic” technology combination according to the proportions of different types of accidents in all traffic accidents in China and obtain the comprehensive crash avoidance rate, as shown in Equation (3). As shown in Equation (1), the expression of “atomic” technology combinations encompasses 14 types of “atomic” technologies. Equation (2) is the calculation method for the unit cost shared by “atomic” technology combinations, with the key in the cost of every “atomic” technology and the number of technologies. Next, the details of the data are introduced.

**Figure 2:**
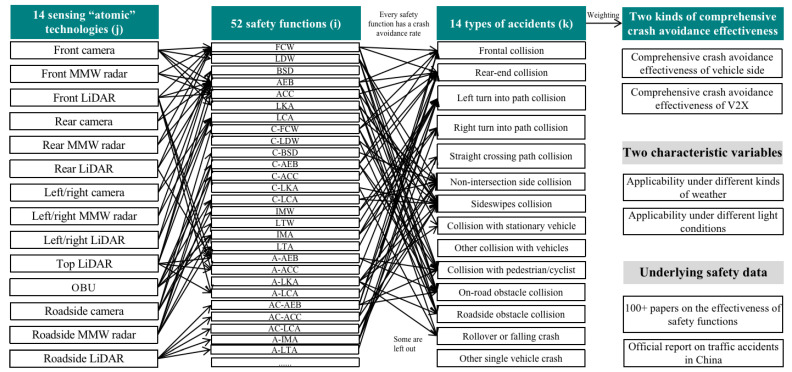
Details of the multivariable coupling model: “Atomic” technology, safety function, accident type, and crash avoidance effectiveness.

More details of the model are shown in Figure 2. The automotive “atomic” technologies include front-facing cameras, front-facing MMW radars, front-facing LiDAR, rear cameras, rear MMW radars, rear LiDAR, side cameras, side MMW radars, side LiDAR, top LiDAR, OBUs, roadside cameras, roadside MMW radars, and roadside LiDAR. All these “atomic” technologies have factored into the applicability under different weather and light conditions. In this paper, “atomic” technologies only focus on the key sensing hardware of autonomous vehicles, and future research will focus on computing chips, steer-by-wire systems, and brake-by-wire systems. There are 26 automotive safety functions, such as front collision warning (FCW), AEB, front cross traffic brake (FCTB), rear collision warning (RCW), rear cross traffic brake (RCTB), ACC, LKA, LDW, BSD, lane change assist (LCA), advanced LKA (A-LKA), and advanced IMA (A-IMA), and there are 26 V2X-based safety functions, such as connected FCW (C-FCW), C-AEB, connected FCTB C-FCTB), C-ACC, C-LKA, connected LCA (C-LCA), connected and advanced LKA (CA-LKA), and connected and advanced IMA (CA-IMA). Every safety function has a corresponding target accident type and crash avoidance rate, for which a database has been created in the previous papers. According to the report on road traffic accidents in China, there are 14 types of accidents, such as head-on collisions, rear-end collisions, side collisions, scrapping, and pedestrian/cyclist collisions. The output crash avoidance effectiveness is divided into the comprehensive crash avoidance effectiveness of autonomous vehicles and of V2X, which can reflect the safety effectiveness of vehicles and roads. The underlying safety-related data are from the safety functions’ crash avoidance effectiveness of nearly one hundred research papers on safety functions and the proportions of accidents listed in the official reports.

(1) $$SensorGroup=\left[x_{1},x_{2},x_{3},x_{4},x_{5},x_{6},x_{7},x_{8},x_{9},x_{10},x_{11},x_{12},x_{13}\right]$$

(2) $$UC_{sensorgroup}=\overset{11}{\underset{j=1}{\sum }}UC_{vehiclesensor,j}*x_{j}+\overset{14}{\underset{j=12}{\sum }}UC_{Roadsensor,j}*x_{j}$$

(3) $$CCAE_{sensorgroup}=\prod_{k=1}^{15}CAE_{k,sensorgroup}\times CP_{k}$$

(4) CAEk,sensorgroup=1−∏j=114(1−CAEk,j)^xj

(5) $$CAE_{k,j}=\left(1-\prod_{i=1}^{52}\left(1-{\mathrm{SF}}_{j,i}\times E_{i}\times {\mathrm{FC}}_{\mathrm{Ik}}\right)\right)\times \left(1-\left(1-W_{j}\right)\times CWP_{w}\right)\times (1-\left(1-L_{j}\right)\times CLP_{L}$$

where: 

SensorGroup is the combination of “atomic” technologies, which is made up of 14 variables (x_j_), x_1_, x_2_, …, x_13_ represent the number of front cameras, front MMW radars, front LiDAR, rear cameras, rear MMW radars, rear LiDAR, left-right cameras, left-right MMW radars, left-right LiDAR, top LiDAR, OBU, roadside cameras, roadside MMW radars, and roadside LiDAR, respectively (If x_1_ = 2, it means there are two front cameras and if x_7_ = 1, it means using four cameras to cover the field of view on the right and left);

*Cost_sensorgroup_* is the cost per kilometer shared by sensorGroup;

UC_vehiclesensor,j_ is the cost per kilometer shared by vehicle-side “atomic” technologies; UC_Roadsensor,j_ is the cost per kilometer shared by roadside “atomic” technologies.

CCAE_Sensorgroup_ represents the comprehensive collision avoidance effectiveness of sensorGroup;

CAE_k,Sensorgroup_ represents the avoidance effectiveness of the accident (k);

CP_k_ is the proportion of the type of accident (k) in all traffic accidents in China;

CAE_k,j_ is the collision avoidance effectiveness of the “atomic technology” (j) to the type of accident (k);

SF_j,i_ represents whether the “atomic” technology (j) can realize the safety function (i) (1 means positive and 0 means negative);

E_i_ is the collision avoidance effectiveness of the safety function (i);

FC_i,k_ represents whether the type of accident (k) is the target accident type of the safety function (i) (1 means positive and 0 means negative);

W_j_ represents whether the “atomic” technology (j) can work in inclement weather (1 means positive and 0 means negative);

L_j_ represents whether the “atomic” technology (j) can work in darkness (1 means positive and 0 means negative);

CWP_w_ is the proportion of traffic accidents in China under inclement weather;

CLP_l_ means the proportion of traffic accidents in China in darkness;

K represents the type of accident, and k = 1, 2, 3, …, 14 means frontal crash, rear-end crash, left-turn crash at a crossing, right-turn crash at a crossing, straight running crash at a crossing, off road obstacle crash, side crash not at a crossing, scrapping, stationary vehicle crash, other vehicle-vehicle crash, pedestrian/cyclist collision, on-road obstacle crash, off road obstacle crash, rollover/rolling/crash, and other vehicle accidents, respectively;

*i* represents 52 safety functions, including AEB, ACC, LKA, C-AEB, C-ACC, and C-LKA.

## 3. Data Description

### 3.1. “Atomic” Technologies

The core sensors of autonomous vehicles include sensors for the external environment and for the vehicles. In this paper, we focus on sensors for external environments, including cameras, MMW radars, and LiDAR. These sensors play different roles when autonomous vehicles run on the road. They can recognize the type, speed, and distance of a nearby object, thus, realizing various safety functions such as AEB, ACC, BSD, LCA, and REB, and therefore avoid accidents to secure the safety of autonomous vehicles [14,15]. V2X has become the autonomous vehicle technology path and development direction to which China has directed significant attention [5,6]. V2X uses roadside cameras, MMW radars, and LiDAR to acquire the speed and distance of the target and transmit key data via OBUs and RSUs to realize safety functions, which are known as connected safety functions, such as C-AEB, C-ACC, C-BSD, C-LCA, and C-REB [9,17,18,19,21]. Generally speaking, in this paper, we focus on a total of 14 vehicle-side and roadside “atomic” technologies as the variables.

The relationship between complete “atomic” technologies and safety functions is shown in Table 1. In this paper, we adopt the following principles: First, with the decoupling of software and hardware, one sensor can realize several safety functions [1,4]. For example, a front camera can identify the front target, range, and parameters to realize FCW, AEB, and ACC, as well as LKA and LDW by recognizing lanes. Second, one safety function can be realized by several sensors [27]. For example, the principle of AEB is to recognize the front vehicle and pedestrian and acquire the range, speed, and other parameters to make necessary breaking, thus, avoiding accidents. Front cameras, MMW radars, and LiDAR can all realize AEB, since they all can recognize the front vehicle and acquire the parameters. Third, different types of sensors can realize different safety functions. For example, a front camera can realize AEB, ACC, LKA, LDW, and other safety functions, while a front MMW radar or a front LiDAR cannot recognize lanes, thus, failing to realize LDW and LKA. Fourth, one “atomic” technology placed in different positions can realize different functions, since the sensor cannot obtain the key parameters to realize safety functions due to directional differences. Front cameras and left/right cameras can realize different safety functions. It is the same with front MMW radars and rear MMW radars. Fifth, roadside cameras, MMW radars, and LiDAR can directly or indirectly transmit data to vehicles to realize connected safety functions, such as C-AEB and C-ACC [28]. Sixth, LiDAR can capture data of 3D point clouds, which is usually installed in premium vehicles. Therefore, LiDAR is assumed to realize safety functions with better crash avoidance effectiveness such as advanced AEB and advanced ACC.

Cameras, MMW radars, and LiDAR have limitations, such as their feasibility under inclement weather and in the darkness [29,30]. The foundation of realizing safety functions is that cameras, radars, and LiDAR can recognize objects surrounding the vehicle. However, cameras and LiDAR cannot work normally under inclement weather (e.g., sandstorms, foggy days, snowy days, etc.). Meanwhile, they cannot perfectly recognize data of the surroundings in the case of poor light, such as nights without streetlights. In this paper, we factored in the feasibility of sensors in the case of inclement weather and darkness. The weather and light conditions under which traffic accidents happened in China are introduced later.

### 3.2. Target Accident Type and Crash Avoidance Rate

In this paper, we cover a total of 26 safety functions that rely on vehicle-side sensors and 26 safety functions that depend on roadside sensors, totaling 52 safety functions, such as AEB and C-AEB, LKA and C-LKA. We have conducted an analysis of global research on the safety function of autonomous vehicles in a systematic and extensive manner and published the results in a previous paper [8]. The crash avoidance effectiveness of safety functions, in this paper, is from the relevant literature and analysis, which can eliminate differences between various research. Safety functions are classified into two categories, namely autonomous vehicle safety functions and V2X safety functions. A function that relies on connected safety functions can have a higher crash avoidance rate than relying on vehicle-side sensors. For example, the crash avoidance rate of C-BSD is 27.1% higher than that of BSD based on vehicle-side sensors [18]. Therefore, the crash avoidance rate of automotive safety functions and connected safety functions can be evaluated with the parameter. Safety functions are also classified into three subcategories, which can provide warning, active control, and enhanced safety functions. According to the simulation research, the crash avoidance rate of LKA is 35%, while that of enhanced safety functions is 51% [31]. If there is the effectiveness of active safety functions, the crash avoidance rate of advanced safety functions could be calculated with the parameter. Complete summaries of crash avoidance effectiveness and target accident types are shown in Table 2 and Table 3.

### 3.3. Traffic Accidents in China

In this paper, we refer to the Report on Traffic Accidents in China issued by the Ministry of Public Security of China in 2020, as the only official data source in China [32]. In 2019, there were 14.27 million road traffic accidents, with 247,646 accident casualties, which is the focus of this paper. Proportions of different types of collisions are important in order to calculate the comprehensive crash avoidance rate of “atomic” technology combinations using Equation (3). There are 14 types of collisions (e.g., sideswipe collision, rear-end collision, and head-on collision). The detailed distribution of different collision types of China’s road traffic crashes is shown in Table A1. To be specific, collisions at intersections (25.0%), non-intersection side collisions (18.5%), and collisions with pedestrians or cyclists (21.6%) account for a high proportion. Therefore, the corresponding sensors can deliver better comprehensive crash avoidance effectiveness. In addition, the distribution of weather conditions and light conditions on road collisions in China in 2019 is shown in Table A2. Specifically, inclement weather accounted for 10.40% of the collisions, and poor light conditions such as dusk and dawns without streetlights accounted for 19.4% of the collisions. Under such circumstances, some sensors cannot work normally.

### 3.4. Cost Sharing

In this paper, the costs of “atomic” technologies refer to the costs of cameras, millimeter-wave radars, and LiDARs obtained based on industry reports of securities institutions and supplier quotations. Vehicle-side cameras and millimeter-wave radars are priced at RMB 990 per unit and RMB 942 per unit, respectively. Vehicle-side LiDARs placed on the front, rear, left, and right are semi-solid-state LiDARs, priced at RMB 4800 per unit. For example, NIO ET7 and CHANA S11 both use semi-solid-state LiDARs. LiDARs placed on the top are mechanical LiDARs, which are generally used in the mechanical LiDAR industry and are priced at RMB 203,667 per unit, far more expensive than semi-solid-state LiDARs. Four sensors are placed on the sides, therefore, the unit cost includes four sensors. As compared with vehicle-side cameras and millimeter-wave radars, roadside cameras and millimeter-wave radars are different and are priced much higher. The costs of roadside cameras, millimeter-wave radars, and LiDARs are RMB 18,000 per unit, RMB 44,500 per unit, and RMB 203,667 per unit, respectively. These data are derived from the costs in the Beijing Autonomous Road Construction Report.

The unit cost (RMB per km) of vehicle-side “atomic” technologies can be obtained based on the life-cycle mileage of the vehicle, as shown in Equation (6). In China, the life-cycle mileage of a vehicle is estimated to be 236,065 km, derived from other studies by our research team [33]. The sharing of the costs of roadside “atomic” technologies has become a new challenging issue. The solution proposed in this paper is that the costs of roadside perception hardware can be shared according to the traffic flow during the life cycle. It mainly involves the costs of roadside “atomic” technologies, the number of atomic technologies required per km of autonomous roads, the average daily traffic flow on the roads, the service life of road equipment, and other parameters. The deployment interval of roadside “atomic” technologies on autonomous roads is about 150–250 m, so the number of facilities required per km is five [5]. According to the monitoring results of 230,000 km of roads in China, the average daily traffic flow on these roads is 14,993 vehicles per day per year [34]. According to national standards, the design life of a road is generally 8–15 years [35]. In this paper, we assume that the service life of roadside facilities is 10 years. Based on the average daily traffic flow on the roads and the service life of roadside facilities, the life-cycle traffic flow of a road can be calculated as 14,993 × 365 × 10 = 54,724,450 trips according to Equation (7). The results of the life-cycle apportioned cost obtained based on Equations (6) and (7) are shown in Table 4.

(6) $$UC_{vehiclesensor,j}=Cost_{vehiclesensor,j}÷VKTL_{vehicle}$$

(7) $$UC_{Roadsensor,j}=Cost_{roadsensor,j}*N÷LU_{road}$$

where:

*UC_vehiclesensor,j_* represents the cost per km shared by the vehicle-side “atomic” technology *j* in the life-cycle mileage of a vehicle;

*VKTL_vehicle_* represents the life-cycle mileage of the vehicle;

*Cost_vehiclesensor,j_* represents the cost of each vehicle-side “atomic” technology *j*;

*UC_Roadsensor,j_* represents the cost shared by the roadside “atomic” technology *j* in the life-cycle traffic flow;

*Cost_roadsensor,j_* represents the cost of each roadside “atomic” technology *j*;

*N* represents the number of roadside “atomic” technologies deployed per km, generally spaced 150–250 m, so here it is assumed that *N* = 5;

*LU_road_* represents the traffic flow of roadside “atomic” technologies during the life cycle.

**Table 4:** Life-cycle shared costs of various “atomic” technologies.

Sensor		Unit Cost (RMB per km)	Cost
Vehicle-side	Frontal camera	0.0042	990
	Frontal millimeter-wave radar	0.0040	942
	Frontal LiDAR (hybrid-solid)	0.0203	4800
	Rear camera	0.0042	990
	Rear millimeter-wave radar	0.0040	942
	Rear LiDAR (hybrid-solid)	0.0203	4800
	Left and right side camera	0.0042 × 4	990 × 4
	Left and right side millimeter-wave radar	0.0040 × 4	942 × 4
	Left and right side LiDAR (hybrid-solid)	0.0203 × 4	4800 × 4
	Top LiDAR (mechanical)	0.8629	203,667
	Vehicle-side OBU	0.0053	1250
Roadside	Roadside camera	0.0016 × 5	18,000 × 5
	Roadside millimeter-wave radar	0.0041 × 5	44,500 × 5
	Roadside mechanical LiDAR	0.0186 × 5	203,667 × 5

## 4. Results

Our findings indicate the avoidance effectiveness and comprehensive collision avoidance effectiveness of various “atomic” technologies placed in different locations in various types of accidents. We also calculated the safety effectiveness of current typical vehicle-side “atomic” technology combinations and roadside “atomic” technology combinations. We calculated the comprehensive collision avoidance effectiveness and corresponding cost per km brought about by tens of thousands of “atomic” technology combinations by enumeration and showed all the situations by drawing a panoramic picture. Based on the panoramic picture, we selected the “atomic” technology combinations with the lowest cost in order to meet the specific safety effect, including the “atomic” technology combinations that rely only on autonomous vehicles and those that rely on V2X. We also selected the optimal “atomic” technology combinations that could achieve safety effects under cost constraints.

### 4.1. Evaluation of the Safety Effect of Various “Atomic” Technologies

**Figure 3:**
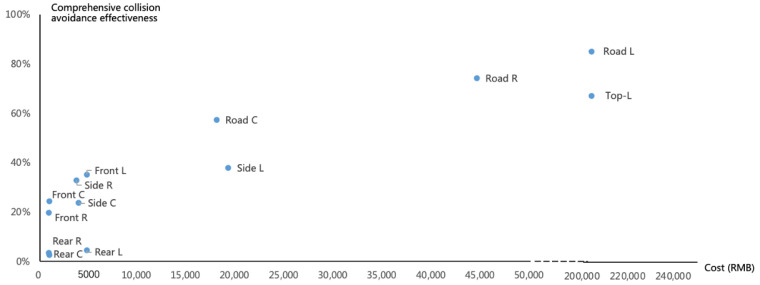
Comprehensive collision avoidance effectiveness and cost of different “atomic” technologies.

The avoidance effectiveness of various “atomic” technologies placed in different locations in various types of accidents and the weighted comprehensive collision avoidance effectiveness obtained based on the proportion of each accident type in China are shown in Figure 3 and Table A3. The weighted comprehensive collision avoidance effectiveness of frontal cameras, frontal millimeter-wave radars, frontal LiDARs, rear cameras, rear millimeter-wave radars, rear LiDARs, side cameras, side millimeter-wave radars, side LiDARs, and top LiDARs are 24.3%, 19.6%, 35.1%, 2.5%, 3.4%, 4.5%, 23.7%, 32.7%, 37.7%, 65.3%, 34.5%, 50.9%, 63.4%, 57.2%, 74.1%, and 82.67%, respectively. The avoidance effectiveness of specific accident types is shown in Table A3. The avoidance effectiveness of a camera placed in front of the vehicle for frontal collision, rear-end collision, non-intersection side collision, sideswipes collision, collision with a stationary vehicle, collision with a pedestrian or cyclist, and obstacle collision is 32.5%, 38.0%, 31.3%, 31.3%, 46.1%, 32.5%, and 49.7%, respectively, and the weighted comprehensive collision avoidance effectiveness is 24.3%. In contrast, the comprehensive collision avoidance effectiveness of frontal millimeter-wave radars is lower, at 19.6%. Although millimeter-wave radars can work normally in darkness and bad weather, their comprehensive collision avoidance effectiveness is still lower than that of frontal cameras because millimeter-wave radars cannot recognize lane lines to achieve LDW/LKA, thus, they are much less effective to avoid side collision accidents. At the same time, the recognition rate of millimeter-wave radars for pedestrians is low, which also reduces the avoidance effectiveness in a collision with a pedestrian or cyclist. The comprehensive collision avoidance effectiveness of frontal LiDARs is 35.1%, higher than that of frontal cameras and frontal millimeter-wave radars, mainly because the introduction of LiDARs can bring enhanced safety functions and improve the effectiveness of the safety functions themselves. If a circle of cameras (two side front cameras, two side rear cameras) is placed in the side perception hardware, the avoidance effectiveness in LTIP, RTIP, SCP, non-intersection side collision and sideswipe collision would be 49.8%, 49.8%, 31.5%, 51.7%, and 51.7% respectively, and the weighted comprehensive collision avoidance effectiveness would be 23.6%. The comprehensive collision avoidance effectiveness of a circle of millimeter-wave radars placed on the side is 37.7%, while that of LiDAR is 37.7%. The 360-degree mechanical LiDARs on the roof of the vehicle can bring a comprehensive collision avoidance effectiveness of 65.3%. From the perspective of cost performance, front cameras, front millimeter-wave radars, side millimeter-wave radars, and side cameras rank in the top four. From the perspective of safety and collision avoidance effectiveness, roadside LiDARs, roadside millimeter-wave radars, top LiDARs, and roadside cameras rank in the top four. From the position in the vehicle, front and side sensors will take priority over rear ones. Compared with vehicle-side sensors, roadside cameras, roadside millimeter-wave radars, and roadside LiDARs can bring a comprehensive collision avoidance effectiveness of 57.2%, 74.1%, and 82.7%, respectively. The safety effect that roadside “atomic” technologies can bring far exceeds that of vehicle-side “atomic” technologies. On the one hand, vehicle-side “atomic” technologies can only be placed in a certain place, on the front, rear, left or right, while roadside ones can cover all directions of the vehicle. On the other hand, the avoidance effectiveness of the networked safety function brought about by roadside sensors is higher than that of the safety function brought about by autonomous vehicles. The comprehensive collision avoidance effectiveness and corresponding cost of each sensor are shown in the following figure. Although roadside sensors can bring higher comprehensive collision avoidance effectiveness, they need sufficient mileage to play their role, and their actual role will depend on the coverage rate. If an autonomous vehicle is driving on a road without roadside sensors, it will not get the safety effect that the roadside sensors will bring. Who will build and pay for these roadside sensors is a challenging question.

### 4.2. Comprehensive Collision Avoidance Effectiveness of Typical “Atomic” Technology Combinations

**Figure 4:**
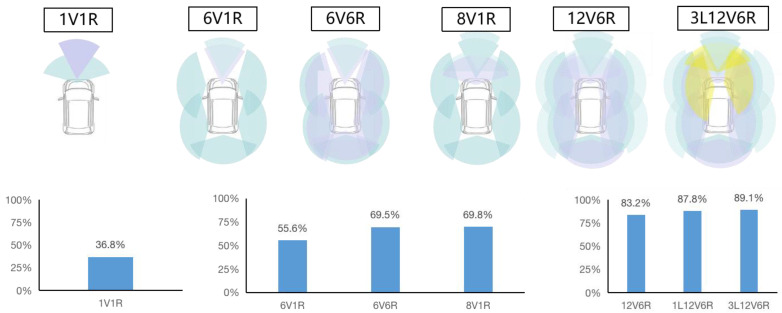
Comprehensive collision avoidance effectiveness of vehicle-side “atomic” technology combinations based on autonomous vehicles (V stands for vision, that is, cameras; Light blue stands for camera, light purple stands for radar, light yellow stands for LiDar).

The safety effectiveness of the current typical “atomic” technology combinations based on autonomous vehicles and those based on V2X is calculated, as shown in Figure 4. The comprehensive collision avoidance effectiveness results of typical vehicle-side “atomic” technology combinations 1V1R (1 camera and 1 mm wave radar), 6V1R, 6V6R, 8V1R, 12V6R, and 3L12V6R are 36.8%, 55.6%, 69.5%, 69.8%, 83.2%, 87.8%, and 89.1%, respectively. Specifically, the 6V6R has five more millimeter-wave radars on the side and rear than the 6V1R, increasing the comprehensive collision avoidance effectiveness from 55.6% to 69.5%, i.e., an increase of 13.9%. As compared with 12V6R, 8V1R has added four cameras and 5 mm wave radars, increasing the comprehensive collision avoidance effectiveness by 13.4%. With an increase in perceptual “atomic” technologies, multiple perceptual redundancy has been achieved on the front, rear, left, and right of the vehicle, and the comprehensive collision avoidance effectiveness has increased, but the safety growth has gradually slowed down. Although LiDARs can result in high safety effects, adding one LiDAR based on 12V6R only results in a small increase in safety performance. From 12V6R to 1L12V6R, the avoidance effectiveness only increases by 4.6% (from 83.2% to 87.8%). If three LiDARs are added, the comprehensive collision avoidance effectiveness only increases from 2.3% to 89.1%, which is unwelcome from the perspective of cost performance.

**Figure 5:**
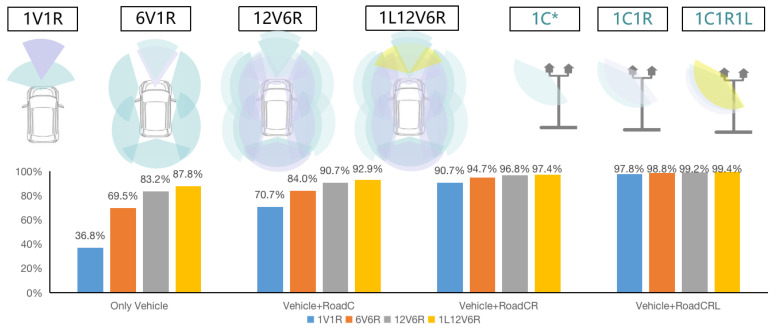
Comprehensive collision avoidance effectiveness of “atomic” technology combinations based on V2X (*C stands for roadside cameras; Light blue stands for camera, light purple stands for radar, light yellow stands for LiDar).

Figure 5 shows the calculation results of the comprehensive collision avoidance effectiveness of typical “atomic” technology combinations based on V2X. We selected 1V1R, 6V1R, 12V6R, and 1L12V6R on the vehicle side, and selected 1C (one roadside camera), 1C1R (one roadside camera and 1 mm wave radar), and 1C1R1L (one roadside camera, 1 mm wave radar, and one LiDAR) on the roadside. The comprehensive collision avoidance effectiveness of a total of 12 permutations and combinations is shown in Figure 5. If we only look at the vehicle side, the comprehensive collision avoidance effectiveness results of 1V1R, 6V1R, 12V6R, and 1L12V6R are 36.8%, 69.5%, 83.2%, and 87.8%, respectively, which are quite different from each other. If we combine vehicle-side and roadside cameras, the comprehensive collision avoidance effectiveness results are 70.7%, 84.0%, 90.7%, and 92.9%, respectively, and the difference between them is reduced. If we combine vehicle-side and roadside cameras and millimeter-wave radars, the comprehensive collision avoidance effectiveness results are 90.7%, 94.7%, 96.8%, and 97.4%, respectively, and the difference between them is further reduced. If we combine vehicle-side and roadside cameras, millimeter-wave radars and LiDARs, the comprehensive collision avoidance effectiveness results are 97.8%, 98.8%, 99.2%, and 99.4%, respectively, and they become very close. When V2X empowers autonomous vehicles, vehicles with low vehicle-side avoidance effectiveness can also achieve high accident avoidance effectiveness. Low-configuration autonomous vehicles can achieve high safety effectiveness when driving on high-configuration autonomous roads. Even if the vehicle-side “atomic” technology combination of 1V1R is combined with roadside cameras and millimeter-wave radars, the comprehensive collision avoidance effectiveness can also reach 90.7%. It is worth noting that autonomous vehicles can obtain a high collision avoidance effectiveness only when driving on autonomous roads equipped with “atomic” technology combinations. On traditional ordinary roads that are not covered by “atomic” technology combinations, they still have a relatively low comprehensive collision avoidance effectiveness.

**Figure 6:**
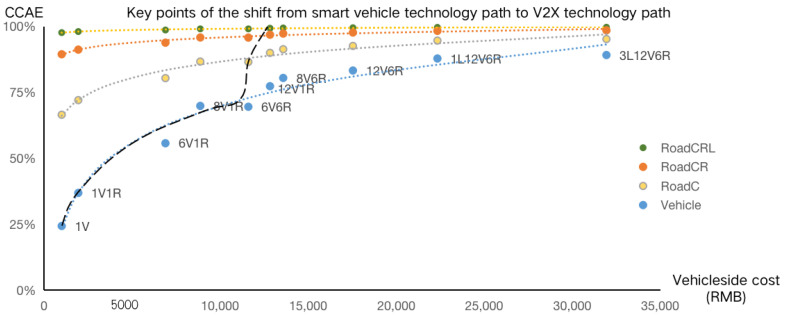
Comprehensive collision avoidance effectiveness and vehicle-side cost of typical “atomic” technology combinations.

In Figure 6, we analyzed the comprehensive collision avoidance effectiveness and vehicle-side cost of “atomic” technology combinations. The horizontal axis is the vehicle-side cost, and the vertical axis is the comprehensive collision avoidance effectiveness (CCAE). The green line at the bottom represents the safety effect and cost of “atomic” technology combinations based on autonomous vehicles. As can be seen, if we upgrade from 1V and 1V1R to 8V1R and 6V6R, the vehicle-side cost required is not high and the safety effectiveness increases significantly. After that, if we upgrade from 6V6R to 12V6R and 1L12V6R, the vehicle-side cost required is further increased, but the increase in safety effects becomes less and less obvious, meaning the cost of improving safety becomes higher and higher. In addition, the yellow, red, and green lines represent the safety effects of the three combinations, i.e., roadside cameras; roadside cameras and millimeter-wave radars; and roadside cameras, millimeter-wave radars, and LiDARs, respectively. As shown, even vehicles with very low configurations can also achieve high comprehensive collision avoidance effectiveness under the empowerment of autonomous roads. This means that there is a significant need to shift from the autonomous vehicle technology path to the V2X technology path in order to improve safety and cost performance, i.e., to achieve higher safety effectiveness at a lower cost. The black curve describes the best route for the transition of the technology path. When rising cost of autonomous vehicles can lead to greatly improved safety, we should choose autonomous vehicles because, in this case, the vehicle-side cost is relatively low. This technology path ensures the safety effectiveness of autonomous vehicles, and also ensures higher safety effectiveness when driving on autonomous roads.

### 4.3. Selection of Optimal “Atomic” Technology Combinations Based on the Comprehensive Collision Avoidance Effectiveness and the Unit Cost

**Figure 7:**
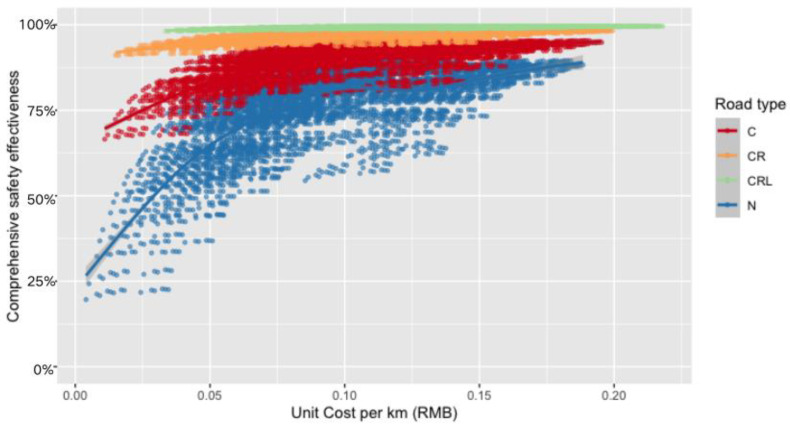
Map of comprehensive collision avoidance effectiveness and life-cycle shared cost of “atomic” technology combinations (unit cost per km).

Figure 7 shows the comprehensive collision avoidance effectiveness and the unit cost shared across the life cycle of all “atomic” technology combinations. We adopted an exhaustive method to assign values to 14 variables in the “atomic” technology combinations, and calculated the comprehensive collision avoidance effectiveness brought about by each “atomic” technology combination and the total unit cost per km of vehicle-side and roadside technologies. The unit cost in the horizontal axis refers to the unit cost per km, which is the sum of the cost per km of vehicle-side sensors apportioned by life-cycle mileage and the cost per km of roadside sensors apportioned by the life-cycle traffic flow. We listed the safety effect and unit cost of all vehicle-side and roadside “atomic” technology combinations to select the optimal “atomic” technology combinations that would meet the corresponding safety requirements or cost requirements. Each point in Figure 7 represents an “atomic” technology combination. Although the horizontal axis is changed to a unit cost per km, the trend is similar to Figure 6. This is mainly because the cost per km of roadside sensors, apportioned by life-cycle traffic flow, is much lower than that of vehicle-side sensors, apportioned by life-cycle mileage. According to this Figure 7, the closer the technology combination is to the upper left corner, the lower the unit cost and the higher the safety effectiveness. “Atomic” technology combinations with fewer vehicle-side sensors and more roadside sensors are closer to the upper left corner, becoming optimal solutions. The closer the technology combination is to the lower right corner, the lower the unit cost and the higher the safety. “Atomic” technology combinations with more vehicle-side sensors and fewer roadside sensors are closer to the lower right corner.

**Figure 8:**
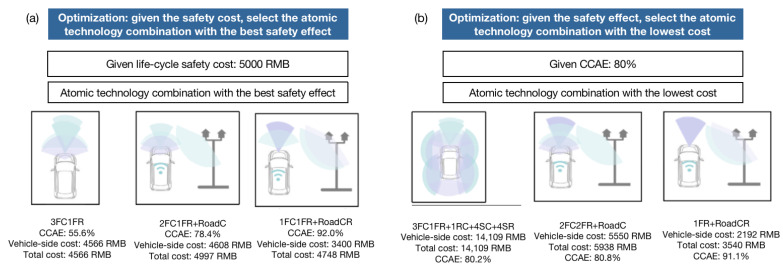
Optimal “atomic” technology combinations under given conditions ((**a**) means the highest safety effectiveness under given cost constraints; (**b**) means the lowest cost under given safety effects; Light blue stands for camera, light purple stands for radar).

Relying on the map of safety and unit cost of “atomic” technology combinations, we can easily obtain the “atomic” technology combination with the highest safety effectiveness under given cost constraints. We limit the life-cycle cost to be within RMB 5000 to find the “atomic” technology combinations with the best safety effect, as shown in Figure 8a. Under autonomous vehicle conditions, the optimal “atomic” technology combination is 3FC1FR, which features a comprehensive collision avoidance effectiveness of 55.6%, a vehicle-side cost of RMB 4566, and a total cost of RMB 4566. Under V2X conditions, the optimal “atomic” technology combination is 1FC1FR + OBU + RoadCR, which features a comprehensive collision avoidance effectiveness of 92.0%, a vehicle-side cost of RMB 3400, and a total cost of RMB 4748. Under RoadC conditions, the optimal “atomic” technology combination is 2FC1FR + OBU + RoadC, which features a comprehensive collision avoidance effectiveness of 78.4, a vehicle-side cost of RMB 4608, and a total cost of RMB 4997. Given the cost limit to be within RMB 5000, as the roadside configuration gradually increases from none, to C, to CR, the comprehensive safety effect of the optimal “atomic” technology combination gradually increases to 55.6%, 78.4%, and 92.0%, respectively.

Relying on the map of safety and unit cost of “atomic” technology combinations, we can also easily obtain the “atomic” technology combination with the lowest cost under given safety effects. We limit the comprehensive collision avoidance effectiveness to be at least 80% to find the “atomic” technology combinations with the lowest cost, as shown in Figure 8b. Under autonomous vehicle conditions, the optimal “atomic” technology combination is 3FC1FR + 1RC + 4SC + 4SR, which features a vehicle-side cost of RMB 14,109, a total cost of RMB 14,109, and a comprehensive collision avoidance effectiveness of 80.2%. Under V2X conditions, the optimal “atomic” technology combination is 1FR + RoadCR, which features a vehicle-side cost of RMB 2192, a total cost of RMB 3540, and a comprehensive collision avoidance effectiveness of 91.1%. Under RoadC conditions, the optimal “atomic” technology combination is 2FC2FR + RoadC, which features a vehicle-side cost of RMB 5550, a total cost of RMB 5938, and a comprehensive collision avoidance effectiveness of 80.8%. Given that the comprehensive collision avoidance effectiveness limit is to be at least 80%, as the roadside configuration gradually increases from none, to C, to CR, both the vehicle-side cost and the life-cycle total cost gradually decrease, with the former decreasing to RMB 14,109, RMB 5550, and RMB 2192, and the latter decreasing to RMB 14,109, RMB 5938, and RMB 3540.

## 5. Discussion

In this paper, we developed an “atomic” technology safety effectiveness evaluation model with the coupling of multiple variables based on “atomic” technologies, safety functions, target accident types, and crash avoidance effectiveness, we evaluated the comprehensive collision avoidance effectiveness of cameras, millimeter-wave radars, and LiDARs placed on the front, rear, side, and top of the vehicle, and cameras, millimeter-wave radars, and LiDARs placed on the roadside, and we also evaluated the comprehensive collision avoidance effectiveness corresponding to different “atomic” technology combinations. In total, 14 types of sensors, 52 safety functions, 14 types of accidents, and the applicability of sensors to bad weather and bad light were considered in the model. In terms of cost, in view of the difficulty in quantifying the cost of vehicle-side and roadside “atomic” technologies, we developed a cost-sharing model based on the traveled distance during the life cycle of vehicles and based on the traffic flow over the life cycle of roads to evaluate the unit cost per km of different “atomic” technology combinations. We quantified the comprehensive collision avoidance effectiveness and the cost of typical vehicle-side and roadside “atomic” technology combinations, drew a panoramic picture of the comprehensive collision avoidance effectiveness and the unit cost per km of all “atomic” technology combinations, and selected the optimal “atomic” technology combination with the lowest cost under given safety requirements and the optimal “atomic” technology combination with the highest safety effect under given cost requirements.

The results clearly show that from the perspective of improving safety effectiveness and reducing usage costs, the V2X technology path is the first choice. The autonomous vehicle path has limitations. As the cost rises, the room for improving safety effectiveness becomes smaller. However, V2X can significantly improve the comprehensive collision avoidance effectiveness at a lower cost. At the same time, due to the existence of roadside “atomic” technologies, in order to achieve the same safety effectiveness, vehicle-side “atomic” technologies can be significantly reduced. Increasing roadside configurations and reducing vehicle-side configurations will become an important and promising direction for the development of autonomous vehicles. V2X has cost and safety advantages, but since autonomous vehicles need to drive on autonomous roads to obtain the benefits of V2X, it is necessary to retain sufficient vehicle-side “atomic” technology combinations to achieve sufficient accident avoidance effectiveness, especially in the initial stage of autonomous road construction.

The problem of selecting optimal “atomic” technology combinations was discussed in this paper. Under the given safety effectiveness, the life-cycle cost of “atomic” technology combinations based on V2X was much lower than that of “atomic” technology combinations based on autonomous vehicles. In this paper, we proposed the optimal “atomic” technology combination with the lowest cost under the assumption of 80% avoidance effectiveness. Under the given cost limit, “atomic” technology combinations with higher roadside configurations can achieve a higher comprehensive collision avoidance effectiveness than “atomic” technology combinations based on autonomous vehicles. In this paper, we also proposed the optimal “atomic” technology combination with the highest safety effectiveness under the constraint of an RMB 5000 life-cycle cost. From the perspective of safety effectiveness and life-cycle shared cost, the results of this paper indicate that V2X will become the future development direction.

This study also points out the directions of future research that need to be studied urgently for promoting the development of the V2X. First, research is needed to quantify the safety impact of penetration rate of autonomous vehicles with different “atomic” technology combinations and the coverage rate of autonomous roads with different “atomic” technology combinations on the annual traffic accident casualty at the country level. In the future, fleets will be composed of traditional vehicles and autonomous vehicles, and roads will consist of traditional roads and autonomous roads. In this paper, we focused on “atomic” technologies. However, the higher-level topic is about the impact of autonomous vehicle deployment and autonomous road deployment on the country’s annual casualties caused by traffic accidents, which would require us to calculate the corresponding economic benefits and total costs from the national level to support further decisions on technology paths. Second, future research should study and formulate corresponding autonomous road policies from the perspective of the government. China has introduced many policies to guide the construction of autonomous roads. Beijing and many other cities have designated special road areas to build autonomous roads in a bid to promote the further development of V2X-based autonomous vehicles. Third, in terms of technology R&D, it is necessary to focus on how to rely on the information of roadside “atomic” technologies to enable the safety functions of autonomous vehicles. In this case, we need to consider time delay, information fusion, reliability, and other scientific issues. Fourth, we need to study the V2X-based business model. There are 5.28 million kilometers of various roads in China. The construction of autonomous roads requires huge costs. Who should pay for the construction costs? How should we recover the costs and achieve profitability in the future?

The limitations of this paper are as follows: First, computing chips, steer-by-wire, brake-by-wire, and some other safety-related basic hardware of autonomous vehicles are not considered in the model, which are also important for supporting safety functions. In fact, the computing power required by chips depends on the size of the information that the vehicle needs to process. When vehicle-side sensors are piled up, chips with large computing power become necessary. Second, in this paper, we fail to consider the impact of more detailed parameters of cameras, millimeter-wave radars, and LiDARs on safety effectiveness. The main modeling logic in this paper is that vehicle-side and roadside “atomic” technologies can support various safety functions, and these safety functions can avoid a certain proportion of target accident types. Due to the lack of data and relevant modeling methods, it is difficult to consider the detailed parameters of various sensors in the research. With more data to be obtained, the model provided in this paper can be further expanded. Third, the effectiveness of the safety functions could be updated if more research on the crash avoidance effectiveness of these safety functions are published. The effectiveness of the same safety function reported by different studies is quite different because studies have used different methods, data and detailed condition parameters. Therefore, the more research results that are considered in a meta-analysis, the more accurately the effectiveness of safety functions can be calculated.

## Figures and Tables

**Table 1:** The relationships between “atomic” technologies and corresponding safety functions.

	Related Safety Functions
Front camera	FCW, AEB, ACC, LDW, LKA
Front MWR	FCW, AEB, ACC
Front Lidar	A-AEB, A-ACC
Rear camera	RCW, REB
Rear MWR	RCW, REB
Rear Lidar	A-REB
Side camera	BSD, LCA, FCTW, FCTB, RCTW, RCTB, LTW, LTA, IMW, IMA
Side MWR	BSD, LCA, FCTW, FCTB, RCTW, RCTB, LTW, LTA, IMW, IMA
Side Lidar	A-LCA, A-FCTB, A-RCTB, A-LTA, A-IMA
Top Lidar	A-AEB, A-ACC, A-LKA, A-REB, A-LCA, A-FCTB, A-RCTB, A-LTA, A-IMA
OBU	None
Road camera	C-FCW, C-FCTW, C-AEB, C-FCTB, C-RCW, C-RCTW, C-REB, C-RCTB, C-ACC, C-LDW, C-LKA, C-BCD, C-LCA, C-IMW, C-LTW, C-IMA, C-LTA
Road MWR	C-FCW, C-FCTW, C-AEB, C-FCTB, C-RCW, C-RCTW, C-REB, C-RCTB, C-ACC, C-BCD, C-LCA, C-IMW, C-LTW, C-IMA, C-LTA
Road Lidar	CA-AEB, CA-ACC, CA-LKA, CA-REB, CA-LCA, CA-FCTB, CA-RCTB, CA-LTA, CA-IMA

**Table 2:** Crash avoidance effectiveness of safety functions.

Safety Function	Crash Avoidance Effectiveness	Supported Sensor
FCW	27.0%	Front camera, front MWR, front LiDar, top LiDar
AEB	45.0%	Front camera, front MWR, front LiDar, top LiDar
ACC	13.9%	Front camera, front MWR, front LiDar, top LiDar
LDW	23.3%	Front camera
LKA	34.2%	Front camera
BSD	30.7%	Side camera, side MWR, side LiDar, top LiDar
LCA	48.2%	Side camera, side MWR, side LiDar, top LiDar
FCTW	27.0%	Side-front camera, side-front MWR, side-front LiDar, top LiDar
FCTB	45.0%	Side-front camera, side-front MWR, side-front LiDar, top LiDar
RCTW	27.0%	Side-rear camera, side-rear MWR, side-rear LiDar, top LiDar
RCTB	45.0%	Side-rear camera, side-rear MWR, side-rear LiDar, top LiDar
RCW	27.0%	Rear camera, rear MWR, rear LiDar, top LiDar
REB	45.0%	Rear camera, rear MWR, rear LiDar, top LiDar
IMW	31.4%	Side-front and side-rear camera/MWR/LiDar, top LiDar
LTW	25.2%	Side-front and side-rear camera/MWR/LiDar, top LiDar
IMA	43.6%	Side-front and side-rear camera/MWR/LiDar, top LiDar
LTA	30.8%	Side-front and side-rear camera/MWR/LiDar, top LiDar
A-AEB	65.7%	Front LiDar, top LiDar
A-ACC	20.2%	Front LiDar, top LiDar
A-LKA	50.0%	Front camera + Front LiDar
A-ALC	70.4%	Side LiDar, top LiDar
A-FCTB	65.7%	Side-front LiDar, top LiDar
A-RCTB	65.7%	Side-rear LiDar, top LiDar
A-REB	65.7%	Rear LiDar, top LiDar
A-IMA	63.6%	Side-front/rear LiDar, top LiDar
A-LTA	57.0%	Side-front/rear LiDar, top LiDar
C-FCW	34.3%	Roadside camera, roadside MWR, roadside LiDar
C-AEB	57.2%	Roadside camera, roadside MWR, roadside LiDar
C-ACC	17.6%	Roadside camera, roadside MWR, roadside LiDar
C-LDW	29.6%	Roadside camera
C-LKA	43.5%	Roadside camera
C-BSD	39.0%	Roadside camera, roadside MWR, roadside LiDar
C-LCA	61.2%	Roadside camera, roadside MWR, roadside LiDar
C-FCTW	34.3%	Roadside camera, roadside MWR, roadside LiDar
C-FCTB	57.2%	Roadside camera, roadside MWR, roadside LiDar
C-RCTW	34.3%	Roadside camera, roadside MWR, roadside LiDar
C-RCTB	57.2%	Roadside camera, roadside MWR, roadside LiDar
C-RCW	34.3%	Roadside camera, roadside MWR, roadside LiDar
C-REB	57.2%	Roadside camera, roadside MWR, roadside LiDar
C-IMW	39.9%	Roadside camera, roadside MWR, roadside LiDar
C-LTW	32.0%	Roadside camera, roadside MWR, roadside LiDar
C-IMA	55.4%	Roadside camera, roadside MWR, roadside LiDar
C-LTA	57.0%	Roadside camera, roadside MWR, roadside LiDar
CA-AEB	83.5%	Roadside LiDar
CA-ACC	25.7%	Roadside LiDar
CA-LKA	63.5%	Roadside camera + Roadside LiDar
CA-ALC	89.4%	Roadside LiDar
CA-FCTB	83.5%	Roadside LiDar
CA-RCTB	83.5%	Roadside LiDar
CA-REB	83.5%	Roadside LiDar
CA-IMA	80.8%	Roadside LiDar
CA-LTA	72.5%	Roadside LiDar

**Table 3:** Target accident matrix of safety functions.

Safety Functions-Collision Types	FCW	AEB	ACC	LDW	LKA	BSD	LCA	FCTW	FCTB	RCTW	RCTB	RCW	REB	IMW	LTW	IMA	LTA	A-AEB	A-ACC	A-LKA	A-ALC	A-FCTB	A-REB	A-IMA	A-LTA
Frontal collision	1	1																1							
Rear-end collision	1	1	1							1	1	1	1					1	1					1	
Left turn into path collision (LTIP)								1	1	1	1			1	1	1	1								1
Right turn into path collision (RTIP)								1	1	1	1			1		1									1
Straight crossing path collision (SCP)														1		1									1
Non-intersection side collision			1	1	1	1	1	1	1	1	1								1	1	1				
Sideswipes collision			1	1	1	1	1	1	1	1	1								1	1	1				
Collision with stationary vehicle	1	1		1	1													1		1					
Other collision with two vehicles																									
Collision with pedestrian or cyclist	1	1												1	1	1	1	1						1	1
On road obstacle collision	1	1																1							
Off road obstacle collision	1	1	1	1	1													1		1					
Rollover or Falling crash																									
Other single vehicle crash																									

Note: Target accidents of 26 safety functions that rely on vehicle-side sensors are listed in the table, while those of 26 connected safety functions that depend on roadside sensors are not included due to the limited space. The target collisions of the other 26 safety functions are the same. For example, the target collision types of C-AEB are the same as AEB, as well as CA-LKA and A-LKA.

## Data Availability

All data are presented in the article. Please contact the corresponding author if you have any question on the data.

## Appendix A

**Table A1:** The detailed distribution of different collision types in road collisions in China.

Collision Type		Collision Number	Proportion
Collision with vehicles	Frontal collision	14,725	5.9%
	Rear-end collision	18,702	7.6%
	Left turn into path collision (LTIP)	21,208	8.6%
	Right turn into path collision (RTIP)	13,705	5.5%
	straight crossing path collision (SCP)	24,415	9.9%
	Non-intersection side collision	45,710	18.5%
	Sideswipes collision	19,079	7.7%
	Collision with stationary vehicle	13,748	5.6%
	Other collision with two vehicles	2452	1.0%
Single vehicle collision	Collision with pedestrian or cyclist	53,558	21.6%
	On road obstacle collision	5016	2.0%
	Off road obstacle collision	5016	2.0%
	Rollover or falling crash	6761	2.7%
	Other single vehicle crash	3551	1.4%
Total collisions		247,646	100%

**Table A2:** The distribution of weather conditions and light conditions in road collisions in China.

Distribution	Category	Proportion of Crashes
Weather condition	Sunny day	74.48%
	Cloudy day	15.12%
	Rainy day	9.58%
	Snowy day	0.44%
	Foggy day	0.27%
	Windy day	0.02%
	Sandstorm	0.01%
	Hail day	0.00%
	Smoggy	0.01%
	Other	0.07%
Light condition	Daytime	58.26%
	Night with streetlight	22.37%
	Night without streetlight	14.72%
	Dusk	2.08%
	Dawn	2.57%

**Table A3:** Avoidance effectiveness of different “atomic” technologies in various types of accidents.

Effectiveness of Sensors	FC	FR	FL	RC	RR	RL	SC	SR	SL	TL	RoadC	RoadR	RoadL
Frontal collision	32.5%	45.0%	58.9%	0.0%	0.0%	0.0%	0.0%	0.0%	0.0%	58.9%	41.3%	57.2%	74.8%
Rear-end collision	38.0%	52.6%	72.7%	32.5%	45.0%	58.9%	0.0%	0.0%	0.0%	65.1%	46.8%	64.7%	78.6%
Left turn into path collision (LTIP)	0.0%	0.0%	0.0%	0.0%	0.0%	0.0%	49.8%	69.0%	78.4%	78.4%	58.4%	80.8%	84.9%
Right turn into path collision (RTIP)	0.0%	0.0%	0.0%	0.0%	0.0%	0.0%	49.8%	69.0%	78.4%	78.4%	58.4%	80.8%	84.9%
straight crossing path collision (SCP)	0.0%	0.0%	0.0%	0.0%	0.0%	0.0%	31.5%	43.6%	57.0%	57.0%	58.4%	55.4%	72.4%
Non-intersection side collision	31.3%	13.9%	28.5%	0.0%	0.0%	0.0%	51.7%	71.5%	80.5%	82.3%	66.7%	86.3%	88.4%
Sideswipes collision	31.3%	13.9%	28.5%	0.0%	0.0%	0.0%	51.7%	71.5%	80.5%	82.3%	66.7%	86.3%	88.4%
Collision with stationary vehicle	46.1%	45.0%	58.9%	0.0%	0.0%	0.0%	0.0%	0.0%	0.0%	58.9%	41.3%	57.2%	74.8%
Other collision with two vehicles	0.0%	0.0%	0.0%	0.0%	0.0%	0.0%	0.0%	0.0%	0.0%	0.0%	41.3%	57.2%	74.8%
Collision with pedestrian or cyclist	32.5%	22.5%	58.9%	0.0%	0.0%	0.0%	0.0%	0.0%	0.0%	58.9%	58.4%	80.9%	86.8%
On road obstacle collision	32.5%	45.0%	58.9%	0.0%	0.0%	0.0%	0.0%	0.0%	0.0%	58.9%	41.3%	57.2%	74.8%
Off road obstacle collision	49.7%	52.6%	72.7%	0.0%	0.0%	0.0%	0.0%	0.0%	0.0%	72.7%	57.9%	64.7%	78.6%
Rollover or falling crash	0.0%	0.0%	0.0%	0.0%	0.0%	0.0%	0.0%	0.0%	0.0%	0.0%	57.9%	64.7%	78.6%
Other single vehicle crash	0.0%	0.0%	0.0%	0.0%	0.0%	0.0%	0.0%	0.0%	0.0%	0.0%	57.9%	64.7%	78.6%
**Weighted comprehensive collision avoidance effectiveness**	24.3%	19.6%	35.1%	2.5%	3.4%	4.4%	23.6%	32.7%	37.7%	65.3%	57.2%	74.1%	82.7%

## References

[1] Zhao F., Song H., Liu Z. (2022). Identification and Analysis of Key Technical Elements and Prospects for Software-Defined Vehicles.

[2] Meng T., Li J., Huang J., Yang D., Zhong Z. (2021). Study on Technical System of Software Defined Vehicles. Automot. Eng..

[3] Rumez M., Grimm D., Kriesten R., Sax E. (2020). An overview of automotive service-oriented architectures and implications for security countermeasures. IEEE Access.

[4] Apostu S., Burkacky O., Deichmann J., Doll G. (2019). Automotive Software and Electrical/Electronic Architecture: Implications for OEMs. https://www.mckinsey.com/industries/automotive-and-assembly/our-insights/automotive-software-andelectrical-electronic-architecture-implications-for-oems.

[5] Liu Z., Song H., Tan H., Hao H., Zhao F. (2022). Evaluation of the Cost of Intelligent Upgrades of Transportation Infrastructure for Intelligent Connected Vehicles. J. Adv. Transp..

[6] Wang B., Han Y., Wang S., Tian D., Cai M., Liu M., Wang L. (2022). A Review of Intelligent Connected Vehicle Cooperative Driving Development. Mathematics.

[7] Liu Z., Song H., Hao H., Zhao F. (2021). Innovation and development strategies of China’s new-generation autonomous vehicles based on 4S integration. Strateg. Study Chin. Acad. Eng..

[8] Tan H., Zhao F., Hao H., Liu Z. (2021). Evidence for the crash avoidance effectiveness of intelligent and connected vehicle technologies. Int. J. Environ. Res. Public Health.

[9] Guglielmi J., Yanagisawa M., Swanson E., Stevens S., Najm W.J. (2017). Safety Benefits of Heavy-Vehicle Crash Warning Applications Based on Vehicle-to-Vehicle Communications.

[10] Jermakian J.S. (2011). Crash avoidance potential of four passenger vehicle technologies. Accid. Anal. Prev..

[11] Fildes B., Keall M., Bos N., Lie A., Page Y., Pastor C., Pennisi L., Rizzi M., Thomas P., Tingvall C. (2015). Effectiveness of low speed autonomous emergency braking in real-world rear-end crashes. Accid. Anal. Prev..

[12] Benmimoun M., Pütz A., Zlocki A., Eckstein L., FISITA (2013). euroFOT: Field Operational Test and Impact Assessment of Advanced Driver Assistance Systems: Final Results. Proceedings of the FISITA 2012 World Automotive Congress.

[13] Andrew L.R., Kiefer J., Meitzner M.R., Flannagan C.A. (2019). Analysis of the Field Effectiveness of General Motors Production Active Safety and Advanced Headlighting Systems.

[14] Riexinger L.E., Sherony R., Gabler H.C. (2019). Residual road departure crashes after full deployment of LDW and LDP systems. Traffic Inj. Prev..

[15] Isaksson-Hellman I., Lindman M. (2016). Evaluation of the crash mitigation effect of low-speed automated emergency braking systems based on insurance claims data. Traffic Inj. Prev..

[16] Schaudt W.A., Bowman D.S., Darrell R.J., Olson R.L., Marinik A., Soccolich S., Joslin S., Toole L., Rice J.C. (2014). Federal Motor Carrier Safety Administration’s Advanced System Testing Utilizing a Data Acquisition System on the Highways (FAST DASH): Safety Technology Evaluation Project# 1 Blindspot Warning (Report No. FMCSA-RRT-13-008).

[17] Scanlon J.M., Sherony R., Gabler H.C. (2017). Injury mitigation estimates for an intersection driver assistance system in straight crossing path crashes in the United States. Traffic Inj. Prev..

[18] Chang J. (2016). Summary of NHTSA Heavy-Vehicle Vehicle-to-Vehicle Safety Communications Research.

[19] Harding J., Powell G.R., Yoon R., Fikentscher J., Doyle C., Sade D., Lukuc M., Simons J., Wang J. (2014). Vehicle-to-Vehicle Communications: Readiness of V2V Technology for Application.

[20] NHTSA (2016). Preliminary Regulatory Impact Analysis: FMVSS No. 150, Vehicle-to-Vehicle Communication Technology for Light Vehicles.

[21] Flannagan C., Leslie A. (2020). Crash Avoidance Technology Evaluation Using Real-World Crash Data.

[22] Parseh M., Asplund F. (2022). New needs to consider during accident analysis: Implications of autonomous vehicles with collision reconfiguration systems. Accid. Anal. Prev..

[23] Esenturk E., Turley D., Wallace A., Khastgir S., Jennings P. (2022). A data mining approach for traffic accidents, pattern extraction and test scenario generation for autonomous vehicles. Int. J. Transp. Sci. Technol..

[24] Čubranić-Dobrodolac M., Švadlenka L., Čičević S., Trifunović A., Dobrodolac M. (2020). Using the Interval Type-2 Fuzzy Inference Systems to Compare the Impact of Speed and Space Perception on the Occurrence of Road Traffic Accidents. Mathematics.

[25] Yu R., Li S. (2022). Exploring the associations between driving volatility and autonomous vehicle hazardous scenarios: Insights from field operational test data. Accid. Anal. Prev..

[26] Hsiang H., Chen K.C., Chen Y.Y. (2022). Development of Simulation-Based Testing Scenario Generator for Robustness Verification of Autonomous Vehicles. Proceedings of the 2022 5th International Conference on Advanced Systems and Emergent Technologies (IC_ASET).

[27] Zhou T., Yang M., Jiang K., Wong H., Yang D. (2020). Mmw radar-based technologies in autonomous driving: A review. Sensors.

[28] Khayyat M., Arrigoni S., Cheli F. (2022). Development and simulation-based testing of a 5G-Connected intersection AEB system. Veh. Syst. Dyn..

[29] Wang Z., Zhan J., Duan C., Guan X., Lu P., Yang K. (2022). A review of vehicle detection techniques for intelligent vehicles. IEEE Trans. Neural Netw. Learn. Syst..

[30] Hafeez F., Sheikh U.U., Alkhaldi N., Al Garni H.Z., Arfeen Z.A., Khalid S.A. (2020). Insights and strategies for an autonomous vehicle with a sensor fusion innovation: A fictional outlook. IEEE Access.

[31] Scanlon J., Kusano K., Sherony R., Gabler H. Potential Safety Benefits of Lane Departure Warning and Prevention Systems in the US Vehicle Fleet. Proceedings of the 24th International Technical Conference on the Enhanced Safety of Vehicles (ESV).

[32] The Ministry of Public Security of the People’s Republic of China (2020). Annual Report on Road Traffic Accidents of the People’s Republic of China.

[33] Zhao F., Liu F., Liu Z., Hao H. (2019). The correlated impacts of fuel consumption improvements and vehicle electrification on vehicle greenhouse gas emissions in China. J. Clean. Prod..

[34] Ministry of Transport of China (2022). Statistical Bulletin of Transport Industry Development in 2021. https://xxgk.mot.gov.cn/2020/jigou/zhghs/202205/t20220524_3656659.html.

[35] (2014). Technical Standards for Highway Engineering.

